# Supplementary material for: In Vitro Nano-Polystyrene Toxicity: Metabolic Dysfunctions and Cytoprotective Responses of Human Spermatozoa
Source: Biology (Basel). 2023 Apr 20;12(4):624. doi: 10.3390/biology12040624 (PMC10136234; doi:10.3390/biology12040624)
Supplement: Supplementary file 1 [file biology-12-00624-s001.zip › authorization patiens.pdf]

Patient Consent Form for Articles Containing Patient Details and/or Images

This form provides consent for MDPI to publish details and/or images from patients. It must be completed prior to publication.

Patient/representative details

Patient name: SARMARTINO STEFANO

If a representative is signing on the patient's behalf:

Name of patient representative: ______________________

Relationship of representative to patient: ______________________

By signing this form, I confirm that I have the authority to represent the patient and provide authorization on their behalf.

Article details

Article title: *In vitro* nano-polystyrene toxicity: metabolic dysfunctions and cytoprotective responses on human spermatozoa.

Journal: Biology.

Authors: Martina Contino, Greta Ferruggia, Stefania Indelicato, Roberta Pecoraro, Elena Maria Scalisi, Giovanni Bracchitta, Jessica Dragotto, Antonio Salvaggio, Maria Violetta Brundo.

Declaration by patient or their representative

I, the patient named above or the patient's representative, have read the abovenamed article in full (including text, figures, and supplementary material) and agree to its publication. I am fully aware of the implications of publication and accept any associated risk. In particular, I understand that, despite anonymization, it is possible that I (or the patient) may be identified based on the details or images contained in the article. While the authors and the publisher will make efforts to minimize this risk, confidentiality cannot be guaranteed.

I understand that the paper will be published online in open access format (using a creative commons CC BY 4.0 license, http://creativecommons.org/licenses/by/4.0), meaning that it can be downloaded, copied and reused without limitation. This include any figures, tables, and supplementary data. The primary audience for the published paper will be healthcare professionals, research academics and students from across the globe.

The final published version may differ from the one submitted to the journal due to minor revisions, changes to style, and reformatting. Publication in the journal mentioned above is not guaranteed and will take place at the discretion of the publisher, and with permission of the Editor-in-Chief (or a qualified Editorial Board member) after a peer review process.

Signing this form does not remove any of my/the patient's statutory rights to privacy. I understand that I may revoke consent at any point prior to publication, but after publication my consent can no longer be withdrawn.

I understand that I/the patient will receive no financial benefit or compensation from publication of the article.

Patient and/or representative signature(s) Sammartino Stefano

Place, date: Ragusa 03/03/2023

Patient Consent Form for Articles Containing Patient Details and/or Images

MDPI

This form provides consent for MDPI to publish details and/or images from patients. It must be completed prior to publication.

Patient/representative details

Patient name: ALESSANDRO SORRENTINO

If a representative is signing on the patient's behalf:

Name of patient representative: ______________________

Relationship of representative to patient: ______________________

By signing this form, I confirm that I have the authority to represent the patient and provide authorization on their behalf.

Article details

Article title: *In vitro* nano-polystyrene toxicity: metabolic dysfunctions and cytoprotective responses on human spermatozoa.

Journal: Biology.

Authors: Martina Contino, Greta Ferruggia, Stefania Indelicato, Roberta Pecoraro, Elena Maria Scalisi, Giovanni Bracchitta, Jessica Dragotto, Antonio Salvaggio, Maria Violetta Brundo.

Declaration by patient or their representative

I, the patient named above or the patient's representative, have read the abovenamed article in full (including text, figures, and supplementary material) and agree to its publication. I am fully aware of the implications of publication and accept any associated risk. In particular, I understand that, despite anonymization, it is possible that I (or the patient) may be identified based on the details or images contained in the article. While the authors and the publisher will make efforts to minimize this risk, confidentiality cannot be guaranteed.

I understand that the paper will be published online in open access format (using a creative commons CC BY 4.0 license, http://creativecommons.org/licenses/by/4.0), meaning that it can be downloaded, copied and reused without limitation. This include any figures, tables, and supplementary data. The primary audience for the published paper will be healthcare professionals, research academics and students from across the globe.

The final published version may differ from the one submitted to the journal due to minor revisions, changes to style, and reformatting. Publication in the journal mentioned above is not guaranteed and will take place at the discretion of the publisher, and with permission of the Editor-in-Chief (or a qualified Editorial Board member) after a peer review process.

Signing this form does not remove any of my/the patient's statutory rights to privacy. I understand that I may revoke consent at any point prior to publication, but after publication my consent can no longer be withdrawn.

I understand that I/the patient will receive no financial benefit or compensation from publication of the article.

Patient and/or representative signature(s) [signature]

Place, date:

02/03/2025 RAGUSA

Patient Consent Form for Articles Containing Patient Details and/or Images

MDPI

This form provides consent for MDPI to publish details and/or images from patients. It must be completed prior to publication.

Patient/representative details

Patient name: Giuseppe Maggiore

If a representative is signing on the patient's behalf:

Name of patient representative: ______________________

Relationship of representative to patient: ______________________

By signing this form, I confirm that I have the authority to represent the patient and provide authorization on their behalf.

Article details

Article title: *In vitro* nano-polystyrene toxicity: metabolic dysfunctions and cytoprotective responses on human spermatozoa.

Journal: Biology.

Authors: Martina Contino, Greta Ferruggia, Stefania Indelicato, Roberta Pecoraro, Elena Maria Scalisi, Giovanni Bracchitta, Jessica Dragotto, Antonio Salvaggio, Maria Violetta Brundo.

Declaration by patient or their representative

I, the patient named above or the patient's representative, have read the abovenamed article in full (including text, figures, and supplementary material) and agree to its publication. I am fully aware of the implications of publication and accept any associated risk. In particular, I understand that, despite anonymization, it is possible that I (or the patient) may be identified based on the details or images contained in the article. While the authors and the publisher will make efforts to minimize this risk, confidentiality cannot be guaranteed.

I understand that the paper will be published online in open access format (using a creative commons CC BY 4.0 license, http://creativecommons.org/licenses/by/4.0), meaning that it can be downloaded, copied and reused without limitation. This include any figures, tables, and supplementary data. The primary audience for the published paper will be healthcare professionals, research academics and students from across the globe.

The final published version may differ from the one submitted to the journal due to minor revisions, changes to style, and reformatting. Publication in the journal mentioned above is not guaranteed and will take place at the discretion of the publisher, and with permission of the Editor-in-Chief (or a qualified Editorial Board member) after a peer review process.

Signing this form does not remove any of my/the patient's statutory rights to privacy. I understand that I may revoke consent at any point prior to publication, but after publication my consent can no longer be withdrawn.

I understand that I/the patient will receive no financial benefit or compensation from publication of the article.

Patient and/or representative signature(s) Giuseppe Maggiore

Place, date:

RAGUSA 02/03/25

Patient Consent Form for Articles Containing Patient Details and/or Images

MDPI

This form provides consent for MDPI to publish details and/or images from patients. It must be completed prior to publication.

Patient/representative details

Patient name: FRANCESCO LA ROSA

If a representative is signing on the patient's behalf:

Name of patient representative: ______________________

Relationship of representative to patient: ______________________

By signing this form, I confirm that I have the authority to represent the patient and provide authorization on their behalf.

Article details

Article title: *In vitro* nano-polystyrene toxicity: metabolic dysfunctions and cytoprotective responses on human spermatozoa.

Journal: Biology.

Authors: Martina Contino, Greta Ferruggia, Stefania Indelicato, Roberta Pecoraro, Elena Maria Scalisi, Giovanni Bracchitta, Jessica Dragotto, Antonio Salvaggio, Maria Violetta Brundo.

Declaration by patient or their representative

I, the patient named above or the patient's representative, have read the abovenamed article in full (including text, figures, and supplementary material) and agree to its publication. I am fully aware of the implications of publication and accept any associated risk. In particular, I understand that, despite anonymization, it is possible that I (or the patient) may be identified based on the details or images contained in the article. While the authors and the publisher will make efforts to minimize this risk, confidentiality cannot be guaranteed.

I understand that the paper will be published online in open access format (using a creative commons CC BY 4.0 license, http://creativecommons.org/licenses/by/4.0), meaning that it can be downloaded, copied and reused without limitation. This include any figures, tables, and supplementary data. The primary audience for the published paper will be healthcare professionals, research academics and students from across the globe.

The final published version may differ from the one submitted to the journal due to minor revisions, changes to style, and reformatting. Publication in the journal mentioned above is not guaranteed and will take place at the discretion of the publisher, and with permission of the Editor-in-Chief (or a qualified Editorial Board member) after a peer review process.

Signing this form does not remove any of my/the patient's statutory rights to privacy. I understand that I may revoke consent at any point prior to publication, but after publication my consent can no longer be withdrawn.

I understand that I/the patient will receive no financial benefit or compensation from publication of the article.

Patient and/or representative signature(s) Francesco La Rosa

Place, date: Ragusa 01/03/2023

Patient Consent Form for Articles Containing Patient Details and/or Images

MDPI

This form provides consent for MDPI to publish details and/or images from patients. It must be completed prior to publication.

Patient/representative details

Patient name: AMICO LUCA

If a representative is signing on the patient's behalf:

Name of patient representative: ______________________

Relationship of representative to patient: ______________________

By signing this form, I confirm that I have the authority to represent the patient and provide authorization on their behalf.

Article details

Article title: *In vitro* nano-polystyrene toxicity: metabolic dysfunctions and cytoprotective responses on human spermatozoa.

Journal: Biology.

Authors: Martina Contino, Greta Ferruggia, Stefania Indelicato, Roberta Pecoraro, Elena Maria Scalisi, Giovanni Bracchitta, Jessica Dragotto, Antonio Salvaggio, Maria Violetta Brundo.

Declaration by patient or their representative

I, the patient named above or the patient's representative, have read the abovenamed article in full (including text, figures, and supplementary material) and agree to its publication. I am fully aware of the implications of publication and accept any associated risk. In particular, I understand that, despite anonymization, it is possible that I (or the patient) may be identified based on the details or images contained in the article. While the authors and the publisher will make efforts to minimize this risk, confidentiality cannot be guaranteed.

I understand that the paper will be published online in open access format (using a creative commons CC BY 4.0 license, http://creativecommons.org/licenses/by/4.0), meaning that it can be downloaded, copied and reused without limitation. This include any figures, tables, and supplementary data. The primary audience for the published paper will be healthcare professionals, research academics and students from across the globe.

The final published version may differ from the one submitted to the journal due to minor revisions, changes to style, and reformatting. Publication in the journal mentioned above is not guaranteed and will take place at the discretion of the publisher, and with permission of the Editor-in-Chief (or a qualified Editorial Board member) after a peer review process.

Signing this form does not remove any of my/the patient's statutory rights to privacy. I understand that I may revoke consent at any point prior to publication, but after publication my consent can no longer be withdrawn.

I understand that I/the patient will receive no financial benefit or compensation from publication of the article.

Patient and/or representative signature(s) [illegible]

Place, date:

Ragusa 1/3/23

Patient Consent Form for Articles Containing Patient Details and/or Images

MDPI

This form provides consent for MDPI to publish details and/or images from patients. It must be completed prior to publication.

Patient/representative details

Patient name: SPADOLA GIOVANNI GABRIELE

If a representative is signing on the patient's behalf:

Name of patient representative: ____________________

Relationship of representative to patient: ____________________

By signing this form, I confirm that I have the authority to represent the patient and provide authorization on their behalf.

Article details

Article title: *In vitro* nano-polystyrene toxicity: metabolic dysfunctions and cytoprotective responses on human spermatozoa.

Journal: Biology.

Authors: Martina Contino, Greta Ferruggia, Stefania Indelicato, Roberta Pecoraro, Elena Maria Scalisi, Giovanni Bracchitta, Jessica Dragotto, Antonio Salvaggio, Maria Violetta Brundo.

Declaration by patient or their representative

I, the patient named above or the patient's representative, have read the abovenamed article in full (including text, figures, and supplementary material) and agree to its publication. I am fully aware of the implications of publication and accept any associated risk. In particular, I understand that, despite anonymization, it is possible that I (or the patient) may be identified based on the details or images contained in the article. While the authors and the publisher will make efforts to minimize this risk, confidentiality cannot be guaranteed.

I understand that the paper will be published online in open access format (using a creative commons CC BY 4.0 license, http://creativecommons.org/licenses/by/4.0), meaning that it can be downloaded, copied and reused without limitation. This include any figures, tables, and supplementary data. The primary audience for the published paper will be healthcare professionals, research academics and students from across the globe.

The final published version may differ from the one submitted to the journal due to minor revisions, changes to style, and reformatting. Publication in the journal mentioned above is not guaranteed and will take place at the discretion of the publisher, and with permission of the Editor-in-Chief (or a qualified Editorial Board member) after a peer review process.

Signing this form does not remove any of my/the patient's statutory rights to privacy. I understand that I may revoke consent at any point prior to publication, but after publication my consent can no longer be withdrawn.

I understand that I/the patient will receive no financial benefit or compensation from publication of the article.

Patient and/or representative signature(s) [illegible]

Place, date: Ragusa 01/03/2023

Patient Consent Form for Articles Containing Patient Details and/or Images

MDPI

This form provides consent for MDPI to publish details and/or images from patients. It must be completed prior to publication.

Patient/representative details

Patient name: LA CIURA STEFANO

If a representative is signing on the patient's behalf:

Name of patient representative: ______________________

Relationship of representative to patient: ______________________

By signing this form, I confirm that I have the authority to represent the patient and provide authorization on their behalf.

Article details

Article title: *In vitro* nano-polystyrene toxicity: metabolic dysfunctions and cytoprotective responses on human spermatozoa.

Journal: Biology.

Authors: Martina Contino, Greta Ferruggia, Stefania Indelicato, Roberta Pecoraro, Elena Maria Scalisi, Giovanni Bracchitta, Jessica Dragotto, Antonio Salvaggio, Maria Violetta Brundo.

Declaration by patient or their representative

I, the patient named above or the patient's representative, have read the abovenamed article in full (including text, figures, and supplementary material) and agree to its publication. I am fully aware of the implications of publication and accept any associated risk. In particular, I understand that, despite anonymization, it is possible that I (or the patient) may be identified based on the details or images contained in the article. While the authors and the publisher will make efforts to minimize this risk, confidentiality cannot be guaranteed.

I understand that the paper will be published online in open access format (using a creative commons CC BY 4.0 license, http://creativecommons.org/licenses/by/4.0), meaning that it can be downloaded, copied and reused without limitation. This include any figures, tables, and supplementary data. The primary audience for the published paper will be healthcare professionals, research academics and students from across the globe.

The final published version may differ from the one submitted to the journal due to minor revisions, changes to style, and reformatting. Publication in the journal mentioned above is not guaranteed and will take place at the discretion of the publisher, and with permission of the Editor-in-Chief (or a qualified Editorial Board member) after a peer review process.

Signing this form does not remove any of my/the patient's statutory rights to privacy. I understand that I may revoke consent at any point prior to publication, but after publication my consent can no longer be withdrawn.

I understand that I/the patient will receive no financial benefit or compensation from publication of the article.

Patient and/or representative signature(s)

Place, date:

Ragusa, 03/03/23

Stefano La Ciura

Patient Consent Form for Articles Containing Patient Details and/or Images

MDPI

This form provides consent for MDPI to publish details and/or images from patients. It must be completed prior to publication.

Patient/representative details

Patient name: CPILICIONE SALVATORE

If a representative is signing on the patient's behalf:

Name of patient representative: ____________________

Relationship of representative to patient: ____________________

By signing this form, I confirm that I have the authority to represent the patient and provide authorization on their behalf.

Article details

Article title: *In vitro* nano-polystyrene toxicity: metabolic dysfunctions and cytoprotective responses on human spermatozoa.

Journal: Biology.

Authors: Martina Contino, Greta Ferruggia, Stefania Indelicato, Roberta Pecoraro, Elena Maria Scalisi, Giovanni Bracchitta, Jessica Dragotto, Antonio Salvaggio, Maria Violetta Brundo.

Declaration by patient or their representative

I, the patient named above or the patient's representative, have read the abovenamed article in full (including text, figures, and supplementary material) and agree to its publication. I am fully aware of the implications of publication and accept any associated risk. In particular, I understand that, despite anonymization, it is possible that I (or the patient) may be identified based on the details or images contained in the article. While the authors and the publisher will make efforts to minimize this risk, confidentiality cannot be guaranteed.

I understand that the paper will be published online in open access format (using a creative commons CC BY 4.0 license, http://creativecommons.org/licenses/by/4.0), meaning that it can be downloaded, copied and reused without limitation. This include any figures, tables, and supplementary data. The primary audience for the published paper will be healthcare professionals, research academics and students from across the globe.

The final published version may differ from the one submitted to the journal due to minor revisions, changes to style, and reformatting. Publication in the journal mentioned above is not guaranteed and will take place at the discretion of the publisher, and with permission of the Editor-in-Chief (or a qualified Editorial Board member) after a peer review process.

Signing this form does not remove any of my/the patient's statutory rights to privacy. I understand that I may revoke consent at any point prior to publication, but after publication my consent can no longer be withdrawn.

I understand that I/the patient will receive no financial benefit or compensation from publication of the article.

Patient and/or representative signature(s)

Place, date: [illegible]

04/03/2023

Patient Consent Form for Articles Containing Patient Details and/or Images

MDPI

This form provides consent for MDPI to publish details and/or images from patients. It must be completed prior to publication.

Patient/representative details

Patient name: [illegible]

If a representative is signing on the patient's behalf:

Name of patient representative: ______________________

Relationship of representative to patient: ______________________

By signing this form, I confirm that I have the authority to represent the patient and provide authorization on their behalf.

Article details

Article title: *In vitro* nano-polystyrene toxicity: metabolic dysfunctions and cytoprotective responses on human spermatozoa.

Journal: Biology.

Authors: Martina Contino, Greta Ferruggia, Stefania Indelicato, Roberta Pecoraro, Elena Maria Scalisi, Giovanni Bracchitta, Jessica Dragotto, Antonio Salvaggio, Maria Violetta Brundo.

Declaration by patient or their representative

I, the patient named above or the patient's representative, have read the abovenamed article in full (including text, figures, and supplementary material) and agree to its publication. I am fully aware of the implications of publication and accept any associated risk. In particular, I understand that, despite anonymization, it is possible that I (or the patient) may be identified based on the details or images contained in the article. While the authors and the publisher will make efforts to minimize this risk, confidentiality cannot be guaranteed.

I understand that the paper will be published online in open access format (using a creative commons CC BY 4.0 license, http://creativecommons.org/licenses/by/4.0), meaning that it can be downloaded, copied and reused without limitation. This include any figures, tables, and supplementary data. The primary audience for the published paper will be healthcare professionals, research academics and students from across the globe.

The final published version may differ from the one submitted to the journal due to minor revisions, changes to style, and reformatting. Publication in the journal mentioned above is not guaranteed and will take place at the discretion of the publisher, and with permission of the Editor-in-Chief (or a qualified Editorial Board member) after a peer review process.

Signing this form does not remove any of my/the patient's statutory rights to privacy. I understand that I may revoke consent at any point prior to publication, but after publication my consent can no longer be withdrawn.

I understand that I/the patient will receive no financial benefit or compensation from publication of the article.

Patient and/or representative signature(s)

Place, date:
3/3/23

Patient Consent Form for Articles Containing Patient Details and/or Images

MDPI

This form provides consent for MDPI to publish details and/or images from patients. It must be completed prior to publication.

Patient/representative details

Patient name: OCCHIPINTI FRANCESCO

If a representative is signing on the patient's behalf:

Name of patient representative: ____________________

Relationship of representative to patient: ____________________

By signing this form, I confirm that I have the authority to represent the patient and provide authorization on their behalf.

Article details

Article title: *In vitro* nano-polystyrene toxicity: metabolic dysfunctions and cytoprotective responses on human spermatozoa.

Journal: Biology.

Authors: Martina Contino, Greta Ferruggia, Stefania Indelicato, Roberta Pecoraro, Elena Maria Scalisi, Giovanni Bracchitta, Jessica Dragotto, Antonio Salvaggio, Maria Violetta Brundo.

Declaration by patient or their representative

I, the patient named above or the patient's representative, have read the abovenamed article in full (including text, figures, and supplementary material) and agree to its publication. I am fully aware of the implications of publication and accept any associated risk. In particular, I understand that, despite anonymization, it is possible that I (or the patient) may be identified based on the details or images contained in the article. While the authors and the publisher will make efforts to minimize this risk, confidentiality cannot be guaranteed.

I understand that the paper will be published online in open access format (using a creative commons CC BY 4.0 license, http://creativecommons.org/licenses/by/4.0), meaning that it can be downloaded, copied and reused without limitation. This include any figures, tables, and supplementary data. The primary audience for the published paper will be healthcare professionals, research academics and students from across the globe.

The final published version may differ from the one submitted to the journal due to minor revisions, changes to style, and reformatting. Publication in the journal mentioned above is not guaranteed and will take place at the discretion of the publisher, and with permission of the Editor-in-Chief (or a qualified Editorial Board member) after a peer review process.

Signing this form does not remove any of my/the patient's statutory rights to privacy. I understand that I may revoke consent at any point prior to publication, but after publication my consent can no longer be withdrawn.

I understand that I/the patient will receive no financial benefit or compensation from publication of the article.

Patient and/or representative signature(s)

Place, date:

Ragusa, 03/03/23

Occhipinti Francesco
