# Supplementary material for: In Vitro Nano-Polystyrene Toxicity: Metabolic Dysfunctions and Cytoprotective Responses of Human Spermatozoa
Source: Biology (Basel). 2023 Apr 20;12(4):624. doi: 10.3390/biology12040624 (PMC10136234; doi:10.3390/biology12040624)
Supplement: Supplementary file 1 [file biology-12-00624-s001.zip › supplementary data.pdf]

Supplementary materials:

Table S1. Summary of motility parameters measured with CASA plugin.

	CTRL	50 nm						100 nm					
		0.1 µg/ml	p	0.5 µg/ml	p	1 µg/ml	p	0.1 µg/ml	p	0.5 µg/ml	p	1 µg/ml	p
Motility (%)	75.63±0.02	67.59±0.03	.70	36.34±0.01	<.001**	22.77±0.01	<.001**	69±0.03	.79	70.35±0.03	.92	71.79±0.01	0.97
VCL	91.9±0.03	75.75±0.04	.91	95.77±0.01	.1	60.68±0.01	.001**	119.34±0.01	.63	72.42±0.02	.88	93.07±0.02	.1
VAP	53.56±0.04	57.52±0.02	.99	65.23±0.02	.51	61.03±0.03	.83	67.58±0.02	.30	53.08±0.03	.1	72.71±0.01	.06
VLS	48.44±0.01	54.24±0.03	.95	59.75±0.03	.46	44.49±0.04	.98	60.01±0.03	.44	50.35±0.01	.99	62.64±0.03	.76
LIN	0.9±0.01	0.93±0.03	.83	0.91±0.02	1	0.74±0.02	<.001**	0.88±0.02	.94	0.94±0.01	.66	0.95±0.01	.4
WOB	0.70±0.01	0.68±0.04	.47	0.67±0.02	.32	1±0.02	<.001**	0.76±0.02	.34	0.74±0.01	.07	0.78±0.01	.06
BCF	27.07±0.02	23.33±0.02	.07	24.51±0.02	.08	27.21±0.02	.42	25.54±0.02	.36	25.78±0.02	.09	23.33±0.04	.12

Data are showed as mean ± standard deviation. Motility is showed as % mean ± standard deviation. Statistically significant differences are indicated with the symbols * ($p<0.05$) and ** ($p<0.01$).

Table S2. Summary of monitored parameters.

	CTRL	50 nm						100 nm					
		0.1 µg/ml	p	0.5 µg/ml	p	1 µg/ml	p	0.1 µg/ml	p	0.5 µg/ml	p	1 µg/ml	p
Intact Membrane (%)	41.15±0.01	27.87±0.02	.744	36.02±0.03	.976	16.52±0.03	.015*	40.31±0.02	.99	39.84±0.03	.99	36.19±0.02	.81
Acrosome Damage (%)	48.14±0.03	55.65±0.03	.423	61.37±0.02	.083	64.54±0.01	.016*	52.65±0.01	.08	59.69±0.03	.1	65.9±0.02	.02*
DNA Fragmentation (%)	36.64±0.03	51.71±0.02	<.001**	52.69±0.01	<.001**	52.5±0.02	<.001**	32±0.03	.87	29.81±0.04	.69	43.28±0.02	.71
Normalized sORP	1.375±0.03	1.525±0.01	.011*	1.53±0.01	.01*	1.8±0.02	.005**	1.585±0.007	.002**	1.59±0.014	.002**	1.65±0.014	.001**
ROS production (%)	1±0.01	9±0.01	.206	17±0.03	.023*	16±0.04	.03*	12±0.02	.01*	9±0.02	.03*	11±0.01	.01*
HSP70 expression (%)	8.79±0.01	57.91±0.02	<.001**	65.33±0.02	<.001**	65.29±0.01	<.001**	11.06±0.001	.25	10.5±0.007	.42	12.32±0.018	.07

Data are showed as % mean ± standard deviation. Statistically significant differences are indicated with the symbols * ($p<0.05$) and ** ($p<0.01$).

Table S3. Summarized data of mitochondrial activity.

	CTRL	50 nm						100 nm					
		0.1 µg/ml	p	0.5 µg/ml	p	1 µg/ml	p	0.1 µg/ml	p	0.5 µg/ml	p	1 µg/ml	p
I class (%)	67±0.007	32±0.002	<.001**	15±0.001	<.001**	9±0.002	<.001**	60±0.004	.12	59±0.005	.07	57±0.006	.08
II class (%)	15±0.003	12±0.001	.22	5±0.003	<.001**	7±0.001	<.001**	17±0.002	.21	15±0.002	.09	20±0.003	.09
III class (%)	11±0.04	17±0.002	.15	20±0.002	.24	14±0.003	.12	16±0.006	.24	11±0.003	.15	12±0.001	.32
IV class (%)	8±0.014	39±0.003	<.001**	70±0.001	<.001**	70±0.002	<.001**	7±0.001	.17	15±0.002	.12	11±0.003	.12

Data are showed as % mean ± standard deviation. Statistically significant differences are indicated with the symbols *($p<0.05$) and **($p<0.01$).
